# Critical roles of airway smooth muscle in mediating deep-inspiration-induced bronchodilation: a big stretch?

**DOI:** 10.1186/s12931-023-02538-8

**Published:** 2023-10-18

**Authors:** Yuto Yasuda, Lu Wang, Pasquale Chitano, Chun Y. Seow

**Affiliations:** 1 Centre for Heart Lung Innovation, St. Paul’s Hospital, Providence Health Care, University of British Columbia, 1081 Burrard Street, Vancouver, BC V6Z 1Y6 Canada; 2 Department of Pathology and Laboratory Medicine, University of British Columbia, Vancouver, BC Canada

**Keywords:** Ex vivo lung mechanics, Lung volume and airway diameter, Strain-induced airway dilation, Airway smooth muscle, Bronchoprotection, Bronchodilation

## Abstract

**Background:**

Deep inspiration (DI) has been shown to induce bronchodilation and bronchoprotection in bronchochallenged healthy subjects, but not in asthmatics. Strain-induced relaxation of airway smooth muscle (ASM) is considered one of the factors responsible for these effects. Other factors include the release or redistribution of pulmonary surfactant, alteration in mucus plugs, and changes in airway heterogeneity.

**Main body:**

The present review is focused on the DI effect on ASM function, based on recent findings from ex vivo sheep lung experiments showing a large change in airway diameter during a DI. The amount of stretch on the airways, when applied to isolated airway rings in vitro, caused a substantial decrease in ASM contractility that takes many minutes to recover. When challenged with a bronchoconstrictor, the increase in pulmonary resistance in the ex vivo ovine lungs is mostly due to the increase in airway resistance.

**Conclusions:**

Although non-ASM related factors cannot be excluded, the large strain on the airways associated with a DI substantially reduces ASM contractility and thus can account for most of the bronchodilatory and bronchoprotective effects of DI.

## Background

An unsettled debate in the field of airway smooth muscle (ASM) and lung function is the role of ASM in mediating bronchodilation induced by a deep inspiration (DI) [1, 2]. The debate emerged subsequent to a comprehensive investigation [3] in isolated bovine, non-asthmatic bronchial segments, in which a stretch on the airway resulting from a DI-mimicking pressure-change was insufficient to reduce ASM contractility and account for the typical bronchodilation observed in non-asthmatic human subjects [4]. Prior to this study, several studies identified significant broncho-relaxation in ex vivo porcine, non-asthmatic airway segments subjected to a transluminal pressure change comparable to that experienced during a DI [5–7]. Etiology behind this discrepancy remains uncertain. In lung slices, oscillatory radial strain has been demonstrated to dilate previously constricted healthy human airways [8], implying that the local airway-parenchyma interdependence could mediate DI-induced bronchodilation. Even though the interdependence does not rely on the presence of ASM in the airways, it is critical in transmitting the distension force from the parenchyma to the airway and ultimately to the ASM. In isolated porcine, non-asthmatic ASM strip preparations, the contractility of the muscle also exhibits a linear decline commensurate with the amplitude of the strain applied to it [9]. Taken together, the aforementioned in vitro studies suggest that oscillatory-strain-induced ASM relaxation may partially account for the observed bronchodilatory response following a DI. However, the relatively large amplitudes and the prolonged oscillation employed in these in vitro studies to achieve physiologically significant broncho-relaxation, has raised doubts regarding the significance of ASM’s role in DI-induced bronchodilation in vivo. In ex vivo sheep lungs, profound bronchodilation has been observed as a consequence of a DI [10]. These findings suggest that extrapulmonary factors, such as neural reflexes, are unlikely to be accountable for DI-induced bronchodilation. However, the studies failed to shed light on the role of ASM in mediating such bronchodilation, as the extent of stretch on the airways (and consequently ASM) remains unknown. To answer the question of whether DI-induced bronchodilation could result from stretching the ASM (thus reducing its contractility), we need to know first how much ASM in intra-lobal airways are stretched during a DI. This brief review is focused on mechanisms related to ASM that may play a role in DI-mediated reduction in bronchodilation. For a broader discussion on other mechanisms, the readers are referred to the recent reviews by Lutchen et al. [11] and Camoretti-Mercado and Lockey [12].

## Why are we interested in the phenomenon of DI-induced bronchodilation?

It is well known that a DI reverses bronchoconstriction in non-asthmatic human subjects [4]. However, this bronchodilatory response to DI is largely absent in asthmatic individuals [13], particularly those with severe asthma [14]. When healthy subjects are prevented from taking deep breaths for a duration of 20–30 min, they develop asthma-like symptoms, which can be alleviated by a DI [15]. DIs administered prior to bronchochallenge in healthy subjects have also been shown to reduce the extent of bronchoconstriction induced by the subsequent challenge [14, 16–18]. This phenomenon is known as the bronchoprotective effect of DI, which is also attenuated in asthmatic individuals. The mechanism underlying DI-induced bronchoprotection may be different from that of bronchodilation. Crimi et al. showed that even in healthy human subjects, the bronchoprotective effect of DIs is absent if the lung function measurement is not preceded by a full lung inflation [19]. This observation was corroborated by studies using isolated porcine bronchial segments [20] and mouse model [18]. What exactly a full lung inflation does to make the lung responsive to the bronchoprotective DI effect is not clear, but the observations suggest that factors affecting lung compliance that may or may not be related to ASM must be involved.

Additionally, in asthmatic subjects, fast re-narrowing of the airways has been observed following DI-induced bronchodilation [21], suggesting that asthmatic ASM may be different from healthy ASM in its response to strain, although shortening velocity and active isometric force of tracheal smooth muscle from human asthmatics was found not to be different from that from non-asthmatics [22, 23]. However, a more recent finding indicates an increase in reactivity of intra-lobal bronchi from human asthmatics compared with those of non-asthmatics [23]. The difference in ASM between asthmatics and non-asthmatics may also lie in their “robustness”, in that the asthmatic muscle’s contractility is less affected by mechanical strain [22], such as that associated with DIs.

The mechanism underlying the DI-induced bronchodilation and bronchoprotection in non-asthmatic subjects is unclear. The diminished or total lack of such response observed by many studies in asthmatics, especially in severe asthmatics, suggests that part of the asthma pathophysiology lies in how a DI alters the lung function. Therefore, it is crucial to elucidate the mechanisms underlying the DI effect, particularly in terms of the role of ASM in lung function. Restoring the DI effect in individuals with asthma could represent a significant breakthrough in asthma treatment.

## How much are intralobular airways distended during a DI?

**Fig. 1:**
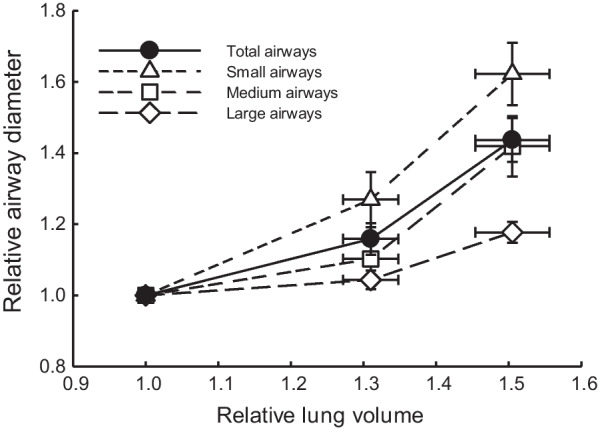
The fractional change in intralobular airway diameter at different lung volumes corresponding to transpulmonary pressures from 5 to 30 cmH_2_O in ex vivo sheep lungs. The airways are grouped into 3 sizes in terms of their diameters, small (< 3 mm), medium (between 3–4 mm), and large (> 4 mm). Reproduced from Dong et al. [24] with permission

In ex vivo sheep lungs, by undertaking a deep inhalation from functional residual capacity (FRC), corresponding to a transpulmonary pressure of 7.5 cm H_2_O, to total lung capacity (TLC), corresponding to a transpulmonary pressure of 40 cm H_2_O, an approximate doubling of ex vivo lung volume has been established [10]. Assuming the lung to be homogeneous and isotropic in its material properties, this volume increase corresponds to an approximate 26% enlargement in airway diameters, as calculated by a scaling factor of 2^1/3^. However, because the lung is neither homogeneous nor isotropic, it is necessary to directly measure airway diameter in intact lungs. To address this matter directly, Dong et al. [24] employed computed tomography (CT) to measure airway diameters and lung volumes at different transpulmonary pressures. Their findings revealed a significant increase in airway diameter and lung volume as the transpulmonary pressure increased from 5 to 30 cmH_2_O, with small airways exhibiting a much greater increase in diameter compared to large airways (Fig. 1). Specifically, with a ~ 50% increase in volume, small airways showed an average diameter increase of ~ 63%, whereas large airways displayed an average increase of ~ 18%. Across all measured airways, the average increase in diameter amounted to ~ 46%. In the context of large airways, Brown et al. observed a ~ 28% increase in canine airway diameter when lung volume doubled from 40 to 80% of maximum volume in unchallenged canine lungs [25]. However, in histamine-challenged lungs, they noted that doubling the volume resulted in a doubling of airway diameter. Similar results were obtained by Sera et al. in mice [26, 27]. An interesting observation from the aforementioned studies is that, despite airway volume representing a tiny fraction of the total lung volume, the fractional increase in the volume of all airways (individually calculated as πr^2^ x airway segment length) exceeded the fractional increase in lung volume. The explanation of this seemingly paradoxical observation could lie in the geometrical disparities between airways and alveoli. Assuming that an airway segment approximates a thin-walled tube, LaPlace law dictates that the tension in the airway wall (T_aw_) equals to the transmural pressure (P_aw_) multiplied by the radius of the airway (r_aw_), i.e., T_aw_ = P_aw_ x r_aw_. In contrast, approximating an alveolus as a thin-walled sphere, the tension in the alveolar wall (T_al_) is determined by the product of the pressure across the alveolar wall (P_al_) times the radius of the alveolus (r_al_) divided by 2, i.e., T_al_ = (P_al_ x r_al_)/2. Considering a static transpulmonary pressure condition where P_aw_ = P_al_, it follows T_aw_ = 2(T_al_ x r_aw_)/r_al_. Given that the alveolar radius is relatively small, approximately 0.1 mm in humans [28], the wall tension in an airway with a 2-mm radius, for example, would be 40 times greater than that in the alveolar wall under the same transpulmonary pressure. Consequently, because of the complex lung structure, airway walls experience substantially greater tension than alveolar walls in the same lung under the same transpulmonary pressure. This phenomenon may elucidate why airways can undergo greater distention compared to lung volume when exposed to distending pressure.

## Can the amount of stretch in ASM seen during a DI reduce ASM contractility?

**Fig. 2:**
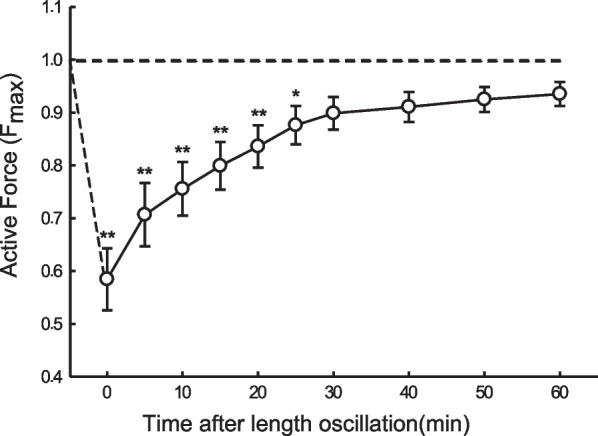
Active force generated by bronchial rings after 3 consecutive stretches at a frequency of 0.25 Hz and strain amplitude of 46% that matched the airway strain observed in intact lungs during a DI. The oscillatory strain was applied just before time zero. *P < 0.05 and **P < 0.01 indicate statistical difference from the maximum isometric force before oscillation (F_max_). Reproduced from Dong et al. [24] with permission

Previous studies have established that oscillatory strain applied to ASM leads to a reduction in its active force, even when the oscillation is applied prior to activation, resulting in a decreased ability to generate force in subsequent contractions [9, 29]. These observations have led to a widespread postulation that the strain exerted on ASM during a DI is responsible for the bronchodilatory and bronchoprotective response. However, before accepting this hypothesis, it is crucial to determine whether the amount of stretch applied to ASM during a DI is significant enough to impact the muscle's contractility. Dong et al. addressed this issue by first quantifying the amount of strain on the airways during a DI and then applying the same level of strain to isolated bronchial rings to evaluate its effect on ASM contractility in an ovine model [24]. As shown in Fig. 2, the 46% average airway strain observed in intact lungs during a DI, when applied to isolated bronchial rings, led to an immediate and substantial reduction in ASM contractility. The depressed force required at least 25 min to recover, indicating that the bronchoprotective effect of DI can have a prolong duration.

**Fig. 3:**
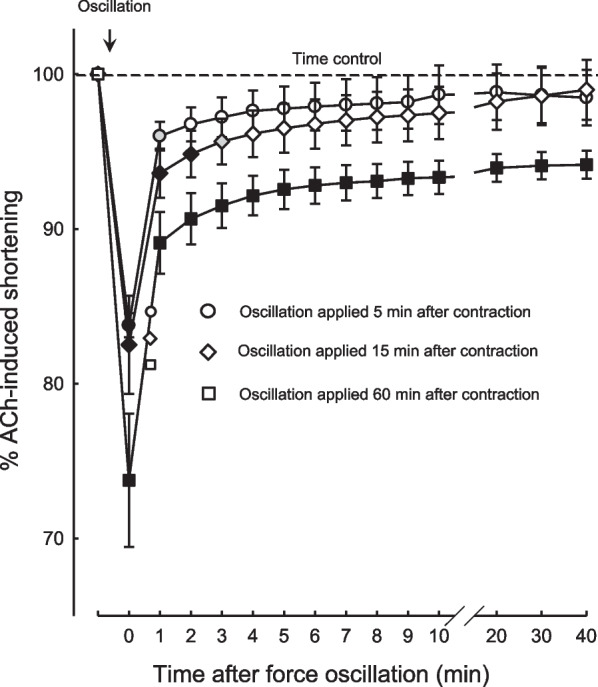
Length relaxation of bronchial rings during an isotonic contraction after 3 cycles of force oscillation (0.25 Hz). The dashed horizontal line represents acetylcholine (ACh)-induced shortening of the ring preparation maintained over time (time control) without interruption by the force oscillation. The oscillation was applied during an isotonic contraction at three different times (5, 15, and 60 min) after the onset of contraction. The solid black symbols represent measurements that are significantly different from the time control with a P value < 0.01, and the gray symbols represent measurements that are significantly different from the time control with a P value < 0.05. The open symbols indicate no difference from time control. Reproduced from Dong et al. [24] with permission

The bronchodilatory effect of DI was investigated by Dong et al. [24] in a different set of experiments. In ovine bronchial rings activated by acetylcholine (ACh), force oscillation at a frequency of 0.25 Hz was applied to the ASM. To mimic the stretch experienced by the bronchus during a DI, the amplitude of the force oscillation was calculated based on the wall tension of the bronchus resulting from a change in the transmural pressure from 5 to 30 cmH_2_O, taking into account the airway diameter according to the LaPlace law. The relaxation in length of the bronchial ring served as an indicator of bronchodilation. Immediately after the force oscillation, there was a large re-lengthening of the ring, followed by re-shortening (Fig. 3). Importantly, the extent of the re-shortening depends on the duration of muscle (ACh-induced) activation before the force oscillation was applied. The longer the muscle had been activated, the less the extent of re-shortening. This observation may have implications for the bronchodilatory effect of DI, suggesting that the longer the airways remain in an actively contracted state, the stronger the bronchodilatory effect of DI.

Based on the data presented in Figs. 2 and 3, it is clear that the amount of stretch experienced by the airways during a DI could be sufficient to account for at least a part of the bronchodilatory and bronchoprotective effect of DI observed in non-asthmatic human subjects [4, 16], but it should be noted that this conclusion is based on observations from ovine bronchial ASM and not that of humans.

## How do we know the reduction in lung resistance after a DI is ASM related?

In isolated sheep lungs, a DI maneuver has been shown to lead to a significant decrease in lung resistance [10]. Lung resistance comprises two components: airway resistance and resistance stemming from the viscoelastic lung parenchymal tissue, also known as tissue resistance. In lungs subjected to bronchochallenge, the increase in airway resistance is primary attributed to the contraction of bronchial smooth muscle, while tissue resistance is largely unrelated to ASM activity. This does not mean that tissue resistance is not a significant component of the lung resistance. In sheep lungs when bronchochallenge caused the lung resistance to double, the airway resistance and tissue resistance each made up about half of the lung resistance when the resistance is measured at 0.25 Hz [30]. Some early studies showed that broncho-challenge caused a significant increase in tissue resistance [31–33]. But a later study showed that this is likely due to broncho-challenge-induced heterogeneity in airway constriction [34].

In the study of Dong et al. the effects of DI on airway and tissue resistance in bronchochallenged sheep lungs were specifically investigated [30]. They found that in these lungs, the airway resistance increased by ~ sixfold after ACh challenge, and about half of this increase was abolished by a DI. On the other hand, tissue resistance was found to be insensitive to ACh challenge, meaning its response to a DI was similar regardless of whether the lungs were challenged or not. This finding in sheep lungs indicates that the part of the lung resistance influenced by ACh resides in the airways, presumably due to the effect of ACh on ASM. The study suggests that the reduction in lung resistance following a DI is primarily ASM-related, and the strain exerted on the airways during a DI is likely responsible for the observed bronchodilation.

## Other factors affected by a DI

From the previous discussion, it is evident that at least a part of the bronchodilatory and bronchoprotective effects of a DI can be attributed to the relaxation of ASM induced by strain. However, it is important to note that a DI may also affect lung resistance through other mechanisms independent of ASM. For instance, it could impact the release or redistribution of pulmonary surfactant, alter mucus plugs, or modify the heterogeneity of airway tone or caliber [11]. Although in healthy ovine lungs we found that airway heterogeneity is not significantly altered by a DI [24]. However, under certain pathological conditions, reducing airway heterogeneity may be an important consequence of a DI. Airway heterogeneity could also be a species-specific issue. We have found heterogeneity in ASM and airway wall area in both asthmatic and non-asthmatic human donor lungs [35]. This finding suggests that there could be heterogeneity in airway constriction in both human asthmatics and non-asthmatics. Given the complexity of lung structure and the presence of numerous cell types, it is conceivable that the bronchodilatory and bronchoprotective effects of DI do not originate from a single locus within the lung.

There are factors known to be influenced by DI but directly or indirectly related to changes in ASM contractility. In asthmatic subjects, the reduction in resistance following DI is inversely associated with the expression of desmin, MLCK, and calponin in bronchial biopsies [36]. The number of mast cells in the ASM area and CD4 positive lymphocytes in the lamina propria are also related to the lack of effectiveness of DI-induced reduction in expiratory resistance in asthmatic subjects [37]. Inhaled glucocorticoids are effective in restoring DI-induced bronchoprotection in mild asthmatic subjects, although their effect is reduced in severe asthmatic subjects [38]. Systemic steroids increase DI-induced bronchodilation in mild to moderate asthmatic subjects [39]. These pieces of evidence suggest that the extent of the effect of DI is inversely related to airway inflammation. In the context of chronic obstructive pulmonary disease (COPD), the bronchodilatory effect of DI is diminished in mild COPD patients [40]. The loss of alveolar attachment observed in COPD is associated with the reduced DI-induced bronchodilation [41].

## Conclusions

**Fig. 4:**
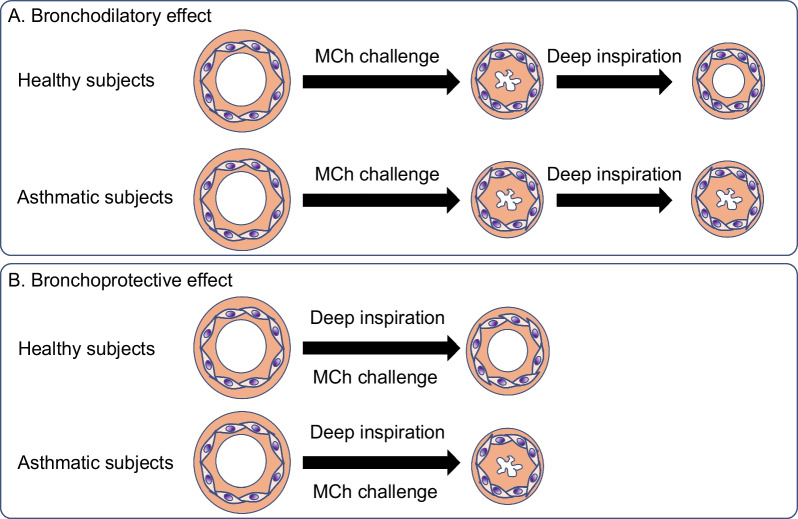
A hypothetical airway-centric view of how a DI, taken after or before bronchochallenge, leads to bronchodilation (**A**) and bronchoprotection (**B**), respectively

Based on recently gathered evidence, the reduction in ASM contractility resulting from DI is emerging as a leading factor believed to mediate the effects of DI. Figure 4 depicts this airway-centric view of the bronchodilatory and bronchoprotective effects of DI. However, it is important to recognize that multiple mechanisms unrelated to ASM could also be involved. Further research is warranted to explore and elucidate these aspects, with the ultimate goal of developing novel drugs that target ASM contractility.

## Data Availability

Data sharing is not applicable to this article. No new data were created or analyzed in this study.
